# C-857-07. Pavement Burns in the Unhoused Community: An Ethical Dilemma of the Built Environment

**DOI:** 10.1093/jbcr/irag033.107

**Published:** 2026-04-06

**Authors:** Ryan Grinnell, Alexander Rowan, Rabia Nizamani, Dustin Dillon

**Affiliations:** Kirk Kerkorian School of Medicine at University of Nevada, Las Vegas, NV; Leon S. Peters Burn Center, Las Vegas, NV; University Medical Center Lions Burn Care Center, Las Vegas, NV; University of Nevada Las Vegas, Department of General Surgery, Las Vegas, NV

## Abstract

**Background:**

When the built environment predictably produces harm, what ethical responsibilities fall to clinicians, public health leaders, and policymakers? In desert cities where ambient temperatures exceed 110°F and pavement surfaces can reach over 150°F, unsheltered individuals are especially vulnerable to severe heat-related injuries such as pavement burns, which frequently necessitate multiple operations and prolonged hospitalization. For providers caring for these patients, the etiology of such injuries raises core ethical questions grounded in nonmaleficence (avoiding harm), beneficence (promoting welfare), and justice (ensuring equitable protection). To address this challenge, we propose an ethical analysis that integrates universal design, an approach to urban planning that creates safe and accessible environments for all people, with the principles of social justice to guide systemic prevention.

**Methods:**

We reviewed literature on universal design with a focus on urban heat islands. A retrospective review of pavement burns at our regional burn center was stratified by housing status. Public health data from the 2024 federally reported Point-in-Time count estimated 7906 people experiencing homelessness in the region, including 4202 unsheltered, within a metropolitan population of 2.4 million (U.S. Census Bureau).

**Results:**

People experiencing homelessness represent only 0.18% of the population, but account for more than 13% of pavement burns at our center. Literature shows the urban heat island effect can raise ambient temperatures by 11°F and disproportionately impacts underserved neighborhoods lacking shade structures and heat-dissipating surfaces.

**Conclusions:**

Pavement burns in extreme-heat cities are a preventable source of morbidity and mortality that disproportionately affects people experiencing homelessness. The foreseeable nature of this harm creates an ethical imperative for collective action. Applying universal design, shade structures, heat-reflective materials, tree cover, and accessible cooling centers, fulfills duties of nonmaleficence and beneficence while advancing social justice. Clinicians, public health leaders, and policymakers share responsibility to advocate for ethically informed urban planning that protects the most vulnerable while benefiting all residents.

**Applicability to Practice:**

Integrating universal design and social justice into community planning requires attention to the dignity and needs of people experiencing homelessness. Continued advocacy, research, and policy development are essential to create safer, more equitable environments.

